# The Faecal Microbiome Analysed from Healthy, Free-Roaming Giraffes (*Giraffa camelopardalis*)

**DOI:** 10.1007/s00284-025-04127-y

**Published:** 2025-02-24

**Authors:** Andri Grobbelaar, Gernot Osthoff, Francois Deacon, Errol D. Cason

**Affiliations:** 1 Department of Animal Sciences, Faculty of Natural and Agricultural Sciences, University of the Free State, PO Box 339, Bloemfontein, 9300 South Africa; 2 Department of Microbiology and Biochemistry, Faculty of Natural and Agricultural Sciences, University of the Free State, PO Box 339, Bloemfontein, 9300 South Africa

## Abstract

Similar to other herbivores, healthy giraffes (*Giraffa camelopardalis*) rely on a variety of symbiotic microorganisms in their digestive systems to break down cellulose and hemicellulose. In this study, we investigate the impact that external stimuli might have on the faecal prokaryote composition of healthy, free-roaming giraffes. Faecal samples were collected from six male and seven female giraffe individuals, over a 2-year period, during the wet and dry seasons, from six locations within the Free State Province, South Africa. Giraffe populations were exposed to one of two feeding practices which included provision of supplemental feed or only naturally available vegetation. Seventeen (17) different prokaryotic phyla, consisting of 8370 amplicon sequence variants (ASVs), were identified from the 13 healthy, adult, free-roaming giraffes included in the study. Overall, the bacterial phyla with the largest relative abundance included Fusobacteria (22%), followed by Lentisphaera (17%) and Cyanobacteria (16%), which included 21 dominant prokaryotic ASVs. The relative abundance of Ruminococcaceae UCG 014 and *Treponema 2* were found to be significantly (*P* < 0.05) higher and *Escherichia* / *Shigella*, *Romboutsia* and *Ruminococcus 1* significantly lower for giraffes receiving supplemental feed compared to natural available vegetation. This is the first study to investigate the composition of the faecal prokaryotic communities of healthy, free-roaming giraffes. The analysis of faecal prokaryotes contributes to the development of non-invasive methods for assessing the nutritional status and identifying health issues in giraffe populations. Ultimately, such advances are beneficial towards the larger-scale conservation, determining nutritional needs and management of other sensitive wildlife species, as well.

**Supplementary Information:**

The online version contains supplementary material available at 10.1007/s00284-025-04127-y.

## Introduction

The rumen microbiome is at the centre of ruminant nutrition, physiology, health and host immunity [1, 2]. The digestive anatomy of ruminants, consisting of the four forestomach compartments (rumen, reticulum, omasum and abomasum), provide a unique and nutrient-rich hosting site for bacteria and protozoa, collectively known as the rumen microbiome [3]. In synergistic symbioses, the rumen microbiome’s enzymatic functioning results in the breakdown of ingested plant fibres, such cellulose and hemicellulose, releasing energy mainly in the form of volatile fatty acids (VFAs) [4–6]. The remaining undigested material, along with a mixture of enzymes, metabolites and microbiota is then excreted in the form of faeces, resulting in a very complex end product of digestive processes occurring inside the animal [7, 8]. Through coevolution between the animal host and the microorganisms, faeces are the final reflector products of the original composition of the diet ingested by the animal and the different needed specialised functions provided by the microorganisms to nurture the animal [8–11]. Faeces can therefore be seen as valuable pockets of information giving an insight to multiple wildlife health factors and the “holobiont” of the animal [12, 13].

In the past, labour intensive and costly techniques such as post-mortem necropsies, captures, blood- and rumen collections were done to gain insight on animal health [14–17]. Such invasive techniques are, however, not appropriate for sensitive species and are usually only performed opportunistically [18]. Faecal collections and specific evaluations could assist in providing a non-invasive and insightful evaluation of the digestive processes within wildlife and biochemical changes in response to internal and/or external stimuli over long time periods [12, 19]. Faeces is directly linked to the digestive system, nutritional- and health status of an individual and can reflect changes in the mammalian host’s diet and metabolism very early [20–22]. Along with assessments in animal welfare such as behaviour and other faecal analyses, i.e., metabolome, hormone and parasite loads, knowledge on the composition of the ruminant microbiome can be used as an effective tool in game management and conservation of sensitive species [1, 23–25].

The tallest animal on earth, the giraffe (*Giraffa camelopardalis*), also relies on this complex gastrointestinal microbial ecosystem [2] and is currently classified as a “vulnerable” species on the International Union for Conservation of Nature (IUCN) Red List [26]. Free-roaming giraffes are selective browsers, utilizing leaves, shoots, fruits, flowers, and even twigs of many different species of trees and shrubs [27] at a height not many other ruminants can reach [28]. An estimated 40% of their daily energy is derived from VFAs, produced by microbial fermentation in the rumen [29], whilst the remaining 60% is from the intestinal digestion of glucose [5]. Their selective diets would most probably result in a distinct microbiome composition.

Even a recent diet change, such as 1 day, was shown to have a noteworthy effect in the human gut microbiota [30]. The mean retention times of particles in giraffes and okapi (*Okapia johnstoni*) have been reported to be approximately 40 h [31, 32] but the verdict is still out on the pace of the change in microbial communities of the reticulo-rumen of the giraffids [15]. Two previous studies from twelve giraffes held in captivity at zoos in North-America [28, 33] and one study from post-mortem procedures performed from seven wild-caught giraffes in South Africa [15] have, to some extent, described the microbiome of giraffes. Two other studies conducted on both healthy and diarrheal giraffe groups in China zoos, also investigated changes in microbial communities [2, 34].

The novelty of the current study lies within investigating the microbiome of free-roaming populations, whilst most previous studies have focused on animals kept in controlled environments, such as zoos and laboratories [8, 24, 35]. Investigations into the microbiome variance within a species, especially from wild populations, could assist in guiding further investigation into the complex relationships between the host microbiome and its external environment [24]. Three hypotheses were evaluated by using 16S rRNA gene-targeted metagenomic sequencing (NGS) on faecal samples [36]. First, we hypothesized that significant differences occur between the prokaryotic composition of faeces analysed from adult, wild giraffe populations receiving supplemental feed (SF) compared to populations only browsing naturally available vegetation (NAV). Second, we hypothesised that differences in the relative abundance of amplicon sequence variants (ASVs) occur between the wet and the dry seasons. Third, we hypothesised that variations in bacterial profiles can be identified for the rumen microbiome composition of the two different giraffe sexes.

## Materials and Methods

### Ethical Statement

Ethical approval was obtained prior to the commencement of the study. The study was approved by the Animal Research Ethics Committee (AREC) of the University of the Free State (UFS) and Nature Conservation (reference numbers: UFS-AED2022/0006). The process was initiated with the formulation of a formal project proposal, which was submitted for review and approval by the scientific review panel, the official animal welfare committee (AREC) of the UFS and the State Veterinarian for Sect.  20 of the Animal Diseases Act, 1984 (Act No. 35 of 1984).

### Sample Collection

Between July 2021 and June 2023, over 300 fresh giraffe faecal samples were collected from six different locations and 13 giraffes in the Free State Province, South Africa. Despite a determined effort to select representative subsets from the various giraffe study populations, achieving a balanced distribution across seasons, feeding practices (i.e., management), locations, ages, and sexes was not possible. To assess the differences in prokaryotic populations related to seasonal dietary changes and dietary supplements, 26 samples (*n* = 26) were selected for further analysis. All giraffes were free-roaming within a 60-km radius of Bloemfontein, located in the Grassland Biome of the Free State Province, South Africa [37]. Feeding practices at the locations involved SF or NAV (Table 1).

**Table 1:** Overview of the samples analysed for 16S rRNA gene-targeted metagenomic sequencing on faecal samples of 13 different individual giraffes provided with supplemental feeding (SF) and those which only fed on the natural available vegetation (NAV) at different locations within the Free State over a 2-year period (2021–2023)

Sample code	Feeding practice	Location (coordinates)	Age class and sex	Date(s) of faecal collections^a^	Defined season
S01	Supplemental Feeding (SF)	**1** (29°05′56″S 26°14′07″E)	Adult Male (I)	03, 18, 31 August 2021	Winter, Dry
S03				09, 18, 28 March 2022	Autumn, Wet
S05				01, 14, 22 June 2022	Winter, Dry
S07				04, 23 November 2022	Summer, Wet
S02				13 February 2022	Summer, Wet
S04				05 and 27 April 2022	Autumn, Wet
S06				11, 24, 25 August 2022	Winter, Dry
S22				07 April 2023	Autumn, Wet
S24				20 March 2023	Autumn, Wet
S09			Adult Male (II)	11 February 2022	N/A^b^
S11				13, 19, 27 April 2022	N/A
S19				07 April 2023	N/A
S08				04, 09, 23 August 2021	N/A
S10				09, 18 March 2022	N/A
S16		**4** (29°03′00″’S 26°13′32″E)	Adult Male	10 March 2023	N/A
S18			Adult Female	10 March 2023	N/A
S28		**5** (29°24′47″S 25°57′30″E)	Adult Female	13 September 2022	N/A
S30				09 October 2022	N/A
S15	Natural Available Vegetation (NAV)	**2** (28°57′16″S 26°23′29″E)	Adult Male	10 March 2023	N/A
S23			Adult Female	22 April 2023	N/A
S14			Adult Female	10 March 2023	N/A
S25		**3** (28°35′50″S 26°25′46″E)	Adult Male	10 April 2023	N/A
M27			Adult Female	10 April 2023	N/A
S26			Adult Female	10 April 2023	N/A
S13		**6** (28°54′47″S 25°50′11″E)	Adult Male	16 February 2023	N/A
S12			Adult Male	16 February 2023	N/A

The faecal samples analysed from a single adult male giraffe (I) at a Location 1, across the wet and dry seasons over the 2-year period are also indicated

^a^If collected on more than one dates, samples were pooled per month

^b^Wet versus dry season comparison was only performed for a single adult male giraffe (indicated as “I”)

As with many studies on free-roaming wild ungulates, our study design was unbalanced with regards to feeding practice, season and sex [38]. The results (*n* = 26) were pooled to distinguish between habitats where SF was provided and those where only NAV was available (Table 1). The SF primarily consisted of manufactured game pellets, which generally contained: crude protein (minimum 100 g/kg), estimated energy (minimum 8 MJ/kg), moisture (maximum 120 g/kg), fat (minimum 30 g/kg), fibre (maximum 250 g/kg), calcium (maximum 10 g/kg), and phosphorus (minimum 4 g/kg) [39, 40]. Supplemental feed (SF) was provided year-round at Locations 1, 4, and 5. Samples were also pooled by month to compare differences in the faecal microbiome during the dry (June to October) and wet (November to May) seasons (*n* = 9) (Table 1).

Samples were collected immediately after defecation to minimize environmental influences on the chemical and biological composition of the faeces. Fresh, wet samples were picked up using gloved hands and placed in plastic storage bags [41], refrigerated at 2 to 4 °C for up to three days, then vacuum-sealed and stored at − 20 °C until analysis, which occurred within 18 months. Plastic bags and prompt cooling or freezing methods were used to prevent changes in the microbial communities, as well as fungal growth, which could alter the prokaryotic composition and relative abundance [41].

### Sample Preparation and Extraction

Faecal samples were thawed, and 0.3 g was taken from the inner portion of each sample using sterile forceps and weighed [33]. Genomic DNA (gDNA) was extracted using the ZymoBIOMICS™ DNA Mini-prep Kit [42] with modifications to the manufacturer’s protocol. These modifications included extending the cell lysis time to 9 min with horizontal shaking on a Vortex Genie® 2 (Scientific Industries Inc, USA). The concentration and purity of the extracted gDNA were measured using a NanoDrop OneC spectrophotometer (Thermo Scientific, USA).

### MiSeq Illumina Sequencing

Extracted gDNA was sent to MRDNA (Shallowater, TX, USA) for NGS using the Illumina MiSeq platform. Per laboratory protocol, the quality and quantity of the extracted gDNA were evaluated with a 2100 Bioanalyzer using 12,000 DNA chips and the Picogreen assay (Invitrogen). The sequencing library was prepared by amplifying an approximately 460 bp region within the V3/V4 hypervariable region of the 16S rRNA gene, followed by the addition of Illumina adapter sequences to the amplicons using specific primers. During a second round of amplification, barcodes were added to distinguish different samples. After gel purification and recovery, the amplified polymerase chain reaction (PCR) products were normalized using the Agencourt AMPure XP Bead Clean-up Kit, pooled, and denatured. The samples were then sequenced on the MiSeq Deep Sequencing System with paired-end reads of 301 bp, using MiSeq v3 reagents.

### Data Analysis and Processing

The raw paired-end sequencing reads were imported into the QIIME2 pipeline (version 2023.2) for further analysis [43]. The initial step involved removing residual sequences from the sequencing experiment using Cutadapt [44], along with the adapter sequences incorporated into the PCR primers during amplification.

Next, quality control was carried out using the DADA2 pipeline [45], which truncates both forward and reverse reads at positions determined by visual inspection of the quality score distributions. Truncation points were chosen at the last position where the 25th percentile of the quality score exceeded a PHRED score of 20. DADA2 then performed dereplication, denoising, chimera identification and removal, and merged the forward and reverse reads to generate representative ASVs.

**Fig. 1:**
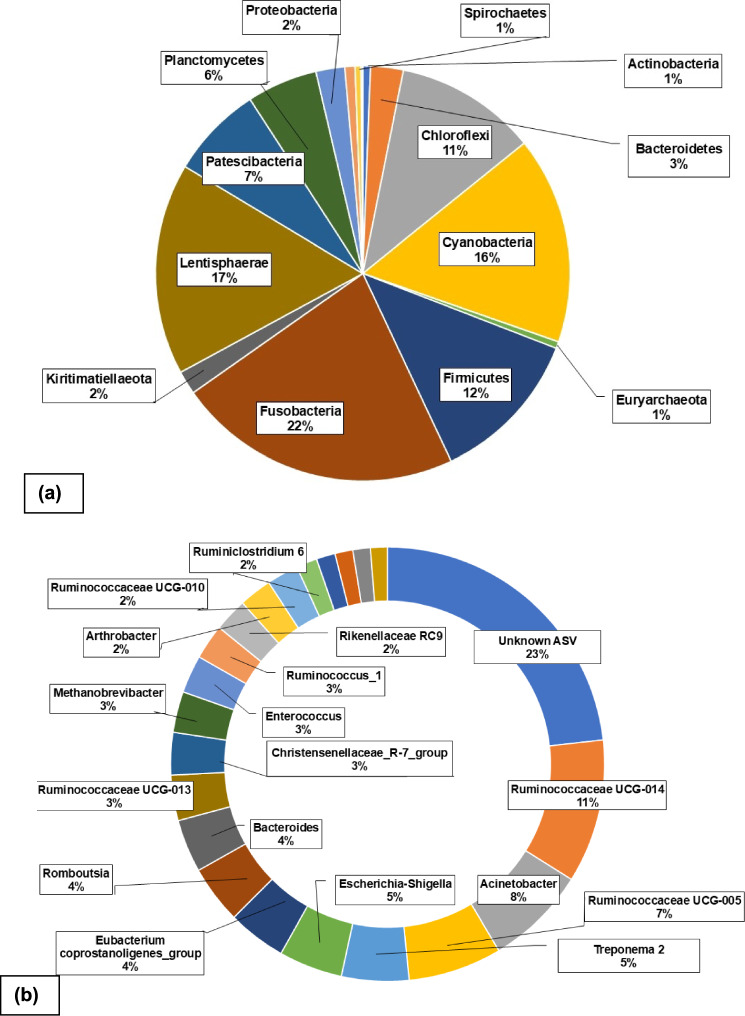
Overall composition (in percentage) of the of the **a** prokaryotic phyla and **b** prokaryotic amplicon sequence variants (based on the 16S ribosomal ribonucleic acid gene) identified from the faecal droppings of thirteen giraffe individuals (*n* = 26) at six different locations situated within the Free State Province, South Africa during a 2-year period (2021–2023)

For taxonomic classification, the q2-feature-classifier plugin was used to train naïve Bayes classifiers on reference datasets using Scikit-learn. For bacterial reads, the pre-formatted SILVA SSU NR 99 full-length reference dataset [46] was used. Classifier training involved extracting amplicon reads from the reference dataset using 16S rRNA V4 primers to improve classification accuracy. Taxonomic classifications of the bacterial reads were then generated using the trained classifiers. The relative abundance table and taxonomic classification were uploaded to MicrobiomeAnalyst 2.0 [47] and MetaboAnalyst 6.0 [48] for further statistical analysis using the available modules. Pie charts were created using Excel [49] to illustrate the overall composition (in percentage) of the prokaryotic at the phylum and ASV level (Fig. 1).

**Fig. 2:**
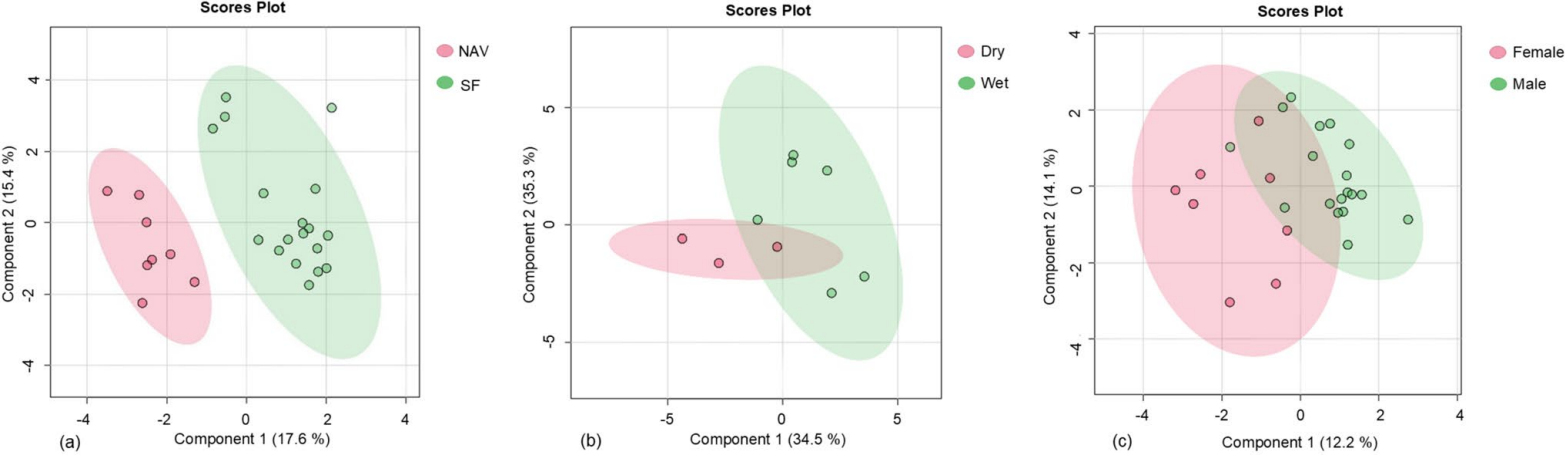
Partial least square discriminant scores plot (PC 1 and 2, with the variation explained in brackets) of faecal prokaryotic profiles (amplicon sequence variants > 1% of total relative abundance) of giraffes **a** provided with supplemental feed (SF) and giraffes only feeding on the natural available vegetation (NAV); **b** of a single giraffe during the wet and dry seasons and **c** for male and female giraffes in free-roaming populations at different locations in the Free State Province, South Africa, over a 2-year period (2021–2023). PC: Principal Component

**Fig. 3:**
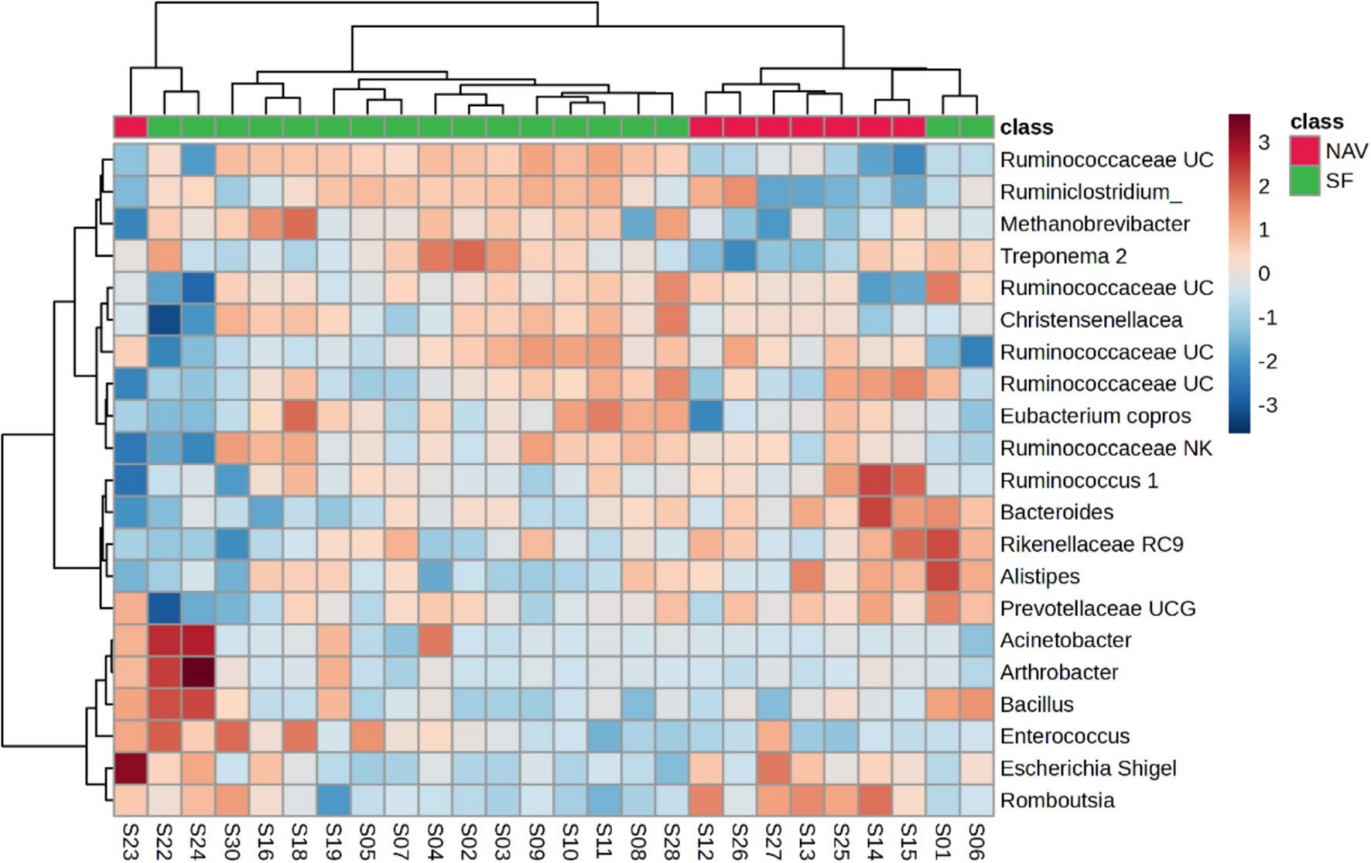
Heatmap (Pearson distance calculation and ward clustering algorithm) of the identified microbiome amplicon sequence variants (> 1% of total abundance, based on the 16S ribosomal ribonucleic acid gene), when comparing data from giraffes receiving supplemental feed and giraffes only feeding on the natural available vegetation from six different locations in the Free State Province, South Africa, over a 2-year period (2021–2023)

**Fig. 4:**
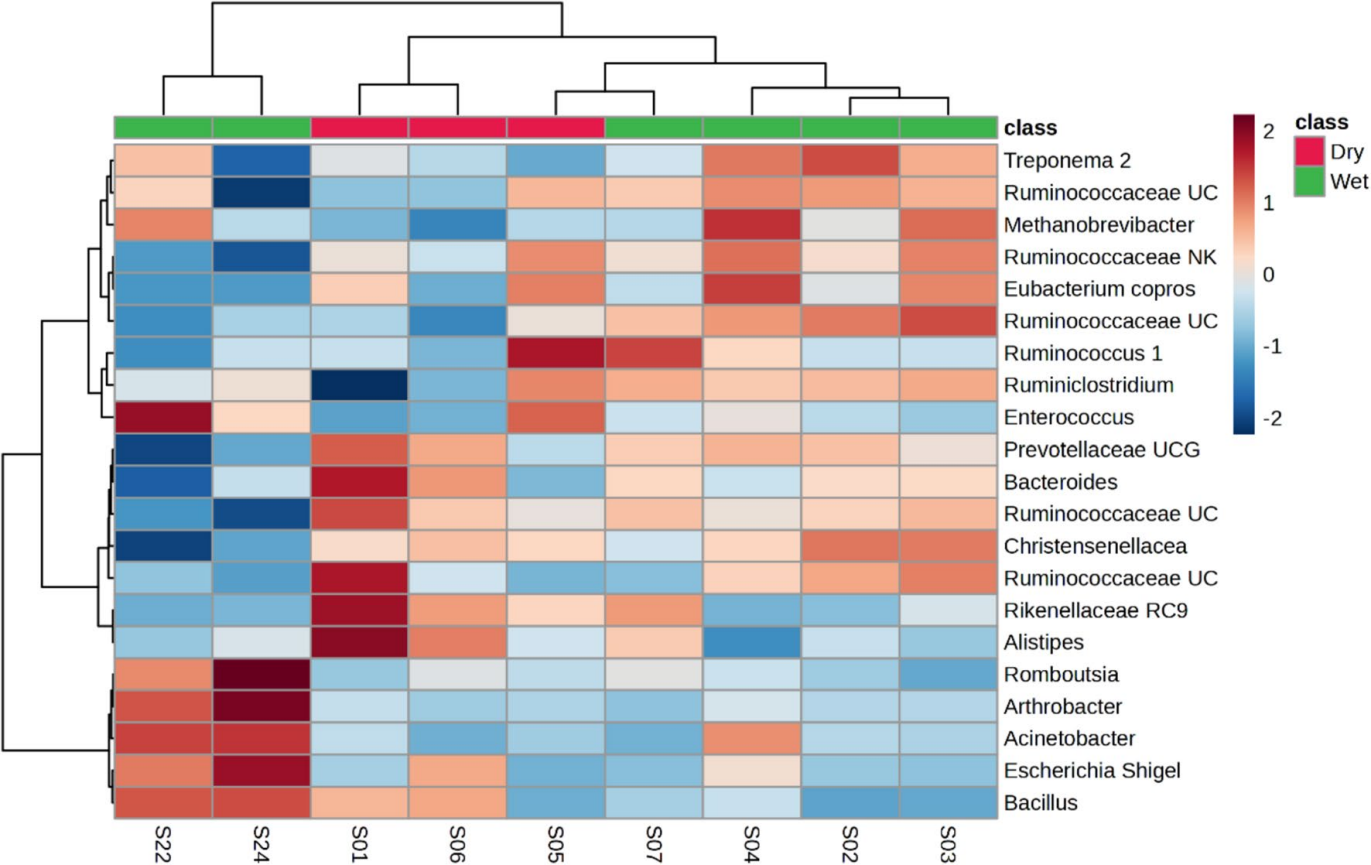
Heatmap (Pearson distance calculation and ward clustering algorithm) of the identified prokaryotic amplicon sequence variants (> 1% of total abundance, based on the 16S ribosomal ribonucleic acid gene), when comparing seasonal variations (wet and dry seasons) from faeces collected from a single adult male giraffe at a Free State location, over a 2-year period (2021–2023)

**Fig. 5:**
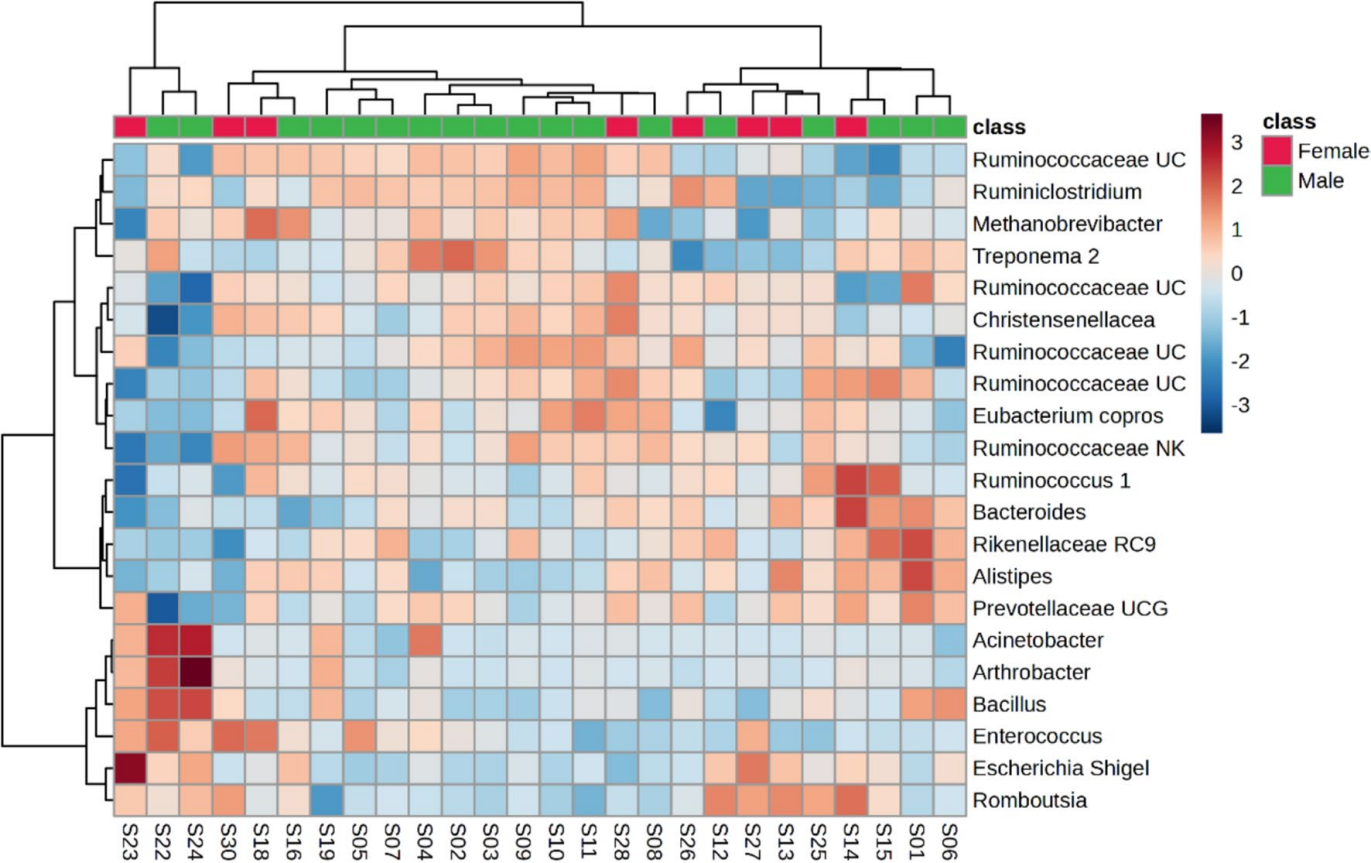
Heatmap (Pearson distance calculation and ward clustering algorithm) of the identified prokaryotic amplicon sequence variants (> 1% of total abundance, based on the 16S ribosomal ribonucleic acid gene), when comparing male and female giraffes’ microbiome profiles from six different locations in the Free State Province South Africa, over a 2-year period (2021–2023)

Before analysis, the data were normalized to the sample median, log-transformed, and auto-scaled. To assess potential confounding effects of the biological information, the processed data were grouped into six subgroups: SF versus NAV (Figs. 2a and 3), wet season versus dry season (Figs. 2b and 4) and male versus female (Figs. 2c and 5). Partial least squares discriminant analysis (PLS-DA) was used to summarize the data by comparing these subgroups. PLS-DA reduces the dimensionality of the input dataset by creating a weighted sum of prokaryote relative abundance, summarizing the variation into a model plane and generating scores. These scores were plotted to provide a visual overview of the samples and their correlations [50].

Fold change analyses were performed and expressed in logarithmic terms (Log^2^) to measure the relative difference between the prokaryotes’ relative abundance between the different subgroups (feeding practice, season and sex) (Table 2). Student’s *t*-test was used to determine whether the observed were statistically significant (*P* > 0.05) (Table 2). Box-whisker plots (Figs. 1, 2, 3 and 4), included in the online Appendix, were generated to illustrate the statistically significant (*P* < 0.05) changes in relative abundance of ASVs between giraffe faeces from SF and NAV conditions.

**Table 2:** Differential faecal prokaryotic amplicon sequence variants (ASVs) (> 1% of total abundance, based on the 16S ribosomal ribonucleic acid gene) identified (with regards to fold change) when comparing three set of subgroups: giraffes provided with SF and giraffes only feeding on the NAV; between wet and dry seasons for the same individual; and between male and female giraffes during the 2-year period (2021–2023)

Prokaryote family	Differential prokaryotic ASVs	SF and NAV^a,b,c^				Wet and Dry Seasons^b,c^			Males and Females^b,c^		
		Fold Change	Log^2^ (FC) (SF and NAV^1^)	Higher (↑) or Lower (↓) in SF^1^	*P*-value	Fold Change	Log^2^ (FC)	Higher (↑) or Lower (↓) in the Wet Season	Fold Change	Log^2^ (FC)	Higher (↑) or Lower (↓) in Females
Moraxellaceae	*Acinetobacter*	0.0260	− 5.268	↑	NS	0.001	− 9.632	↑	0.026	− 5.270	↓
Rikenellaceae	*Alistipes*	NA	NA	NA	NA	4.841	2.275	**↓**	NA	NA	NA
Micrococcaceae	*Arthrobacter*	0.049	− 4.350	↑	NS	0.007	− 7.102	↑	0.053	− 4.248	↓
Bacillaceae	*Bacillus*	0.169	− 2.565	↑	NS	0.323	− 1.630	↑	0.180	− 2.473	↓
Enterococcaceae	*Enterococcus*	0.359	− 1.480	↑	NS	NA	NA	NA	2.417	1.274	↑
Enterobacteriaceae	*Escherichia / Shigella*	20.017	4.323	↓	0.003	0.413	− 1.277	↑	16.283	4.025	↑
Methanobacteriaceae	*Methanobrevibacter*	0.219	− 2.189	↑	0.002	0.463	− 1.110	↑	NA	NA	NA
Prevotellaceae	Prevotellaceae UCG 004	NA	NA	NA	NA	2.040	1.029	**↓**	NA	NA	NA
Rikenellaceae	Rikenellaceae RC9	NA		NA	NA	4.059	2.021	**↓**	NA	NA	NA
Peptostreptococcaceae	*Romboutsia*	5.421	2.439	↓	0.0001	0.459	− 1.122	↑	3.058	1.613	↑
Ruminococcaceae	Ruminococcaceae UCG 010	NA	NA	NA	NA	2.661	1.412	**↓**	NA	NA	NA
Ruminococcaceae	Ruminococcaceae UCG 013	NA	NA	NA	NA	0.412	− 1.280	↑	NA	NA	NA
Ruminococcaceae	Ruminococcaceae UCG 014	0.156	− 2.680	↑	0.0002	NA	NA	NA	NA	NA	NA
Ruminococcaceae	*Ruminococcus 1*	2.024	1.017	↓	NS	NA	NA	NA	NA	NA	NA
Treponemataceae	*Treponema 2*	0.249	− 2.008	↑	0.0099	0.299	− 1.74	↑	0.195	− 2.357	↓

^a^NAV: natural available vegetation; *SF* supplemental feeding

^b^NS: Prokaryote genus was classified as not-significant with the student’s *t*-test analyses (*P* > 0.05)

^c^*NA* Not applicable as the genus was not identified as differential between subgroups

## Results

### Population

A total of 26 faecal samples from 13 different free-roaming giraffes were included in this study. The composition of the subgroups for analysis (feeding practice, season and sex) is described (Table 1).

### Overview of the Microbiome Data and Subgroup Comparisons

Seventeen (17) different prokaryotic phyla and 8370 ASVs were identified from the faecal droppings from 13 giraffe individuals over the 2-year period (Fig. 1a). The largest abundance was calculated for the bacteria phyla Fusobacteria (22%), followed by Lentisphaera (17%) and Cyanobacteria (16%) (Fig. 1a), which included 21 dominant prokaryotic ASV (more than 1% of total sequences) such as *Acinetobacter*, *Alistipes*, *Arthrobacter*, *Bacillus*, Bacteroides, Christensenellaceae R 7, *Enterococcus*, *Escherichia* / *Shigella*, *Eubacterium coprostanoligenes*, *Methanobrevibacter*, Prevotellaceae UCG 004, Rikenellaceae RC9, *Romboutsia*, *Ruminiclostridium 6*, Ruminococcaceae NK4A214, Ruminococcaceae UCG 005, Ruminococcaceae UCG 010, Ruminococcaceae UCG 013, Ruminococcaceae UCG 014, *Ruminococcus 1* and *Treponema 2* (Fig. 1b).

Clear separate profiles between the data obtained from the SF and NAV giraffe samples were seen on the PLS-DAs scores plot (Fig. 2a). Higher relative abundance of ASVs such as *Acinetobacter*, *Arthrobacter*, *Bacillus*, *Enterococcus*, *Methanobrevibacter*, Ruminococcaceae UCG 014 and *Treponema 2* (Fig. 3), were noted for giraffes receiving SF, compared to the NAV subgroup of which Ruminococcaceae UCG 014 and *Treponema 2* were found to be significantly (*P* < 0.05) higher (Table 2). *Escherichia* / *Shigella*, *Romboutsia* and *Ruminococcus 1* were found to be significantly (*P* < 0.05) lower in relative abundance for giraffes receiving SF (Table 2).

Separate faecal bacterial profiles were found across the wet and dry seasons, identified from a single adult male giraffe (indicated as “I” in Table 1 and in Fig. 2b). Higher relative abundance of ASVs such as *Acinetobacter*, *Arthrobacter*, *Bacillus*, *Escherichia* / *Shigella*, *Methanobrevibacter*, *Romboutsia*, Ruminococcaceae UCG 013 and *Treponema 2* (Table 2, Fig. 4) were noted for the wet season, compared to the dry season, whilst lower relative abundance was calculated for *Alistipes*, Prevotellaceae UCG 004, Rikenellaceae RC9 and Ruminococcaceae UCG 010. No differences in relative abundance were, however, recorded as significant (Table 2).

Variation in bacterial profiles was also identified for the microbiome from faecal samples analysed from the six males and seven females (Fig. 2c). Non-significant (*P* > 0.05) differences, such as higher relative abundance of *Enterococcus*, *Escherichia* / *Shigella*, *Romboutsia* and lower relative abundance of *Alistipes*, *Arthrobacter*, *Bacillus* and *Treponema 2* (Table 2 and Fig. 5) were noted for females when compared to males.

## Discussion

The small sample size of this study necessitates its classification as a pilot investigation aimed at characterizing the prokaryotic composition and identifying differences between subgroups. Specifically, the study explores whether bacterial composition can serve as a tool to investigate the effects of sex, feeding practices, and seasonal dietary variation on the faecal composition of healthy, free-roaming giraffes. Future research should incorporate larger and more diverse sample sizes to enable the development of robust models for more comprehensive data interpretation and stronger conclusions.

During this study seventeen different prokaryotic phyla were detected, which included six more phyla than previously described from the amplicon sequencing of the 16S rRNA gene done on giraffe faecal droppings from an American zoo [28]. The six additional phyla included Chloroflexi, Euryarchaeota, Fusobacteria, Kiritimatiellaeota, Patescibacteria and Synergistetes (Fig. 1a). In another study on wild-caught giraffe, 21 phyla were found in the rumen of euthanized giraffe, mostly dominated by *Firmicutes* (50%), *Bacteriodetes* (30%) and *Proteobacteria* (4%) [15]. During the current study the largest abundances (of combined samples) were calculated for the bacteria phyla Fusobacteria (22%), followed by Lentisphaera (17%) and Cyanobacteria (16%) (Fig. 1a). In two other studies both conducted on the faeces from captive giraffe the mean bacterial community composition was dominated by *Firmicutes* (36% and 44%), *Bacteroidetes* (18% and 32%) and *Spirochaetes* (12 and 22%) [28, 33].

Both previous studies conducted on giraffes held in captivity at China zoos demonstrated dramatic decreases in alpha diversity, accompanied by distinct alterations in taxonomic compositions of gut bacterial communities for diarrheal giraffes [2, 34]. In comparison with the healthy zoo-housed giraffes, specifically the proportion of Proteobacteria in the diarrheal giraffes was increased, whilst Bacteroidetes, Firmicutes, Tenericutes, and Spirochaetes were significantly decreased in diarrheal giraffes [34]. The same dramatic decrease in relative abundance of two bacterial phyla, including eight genera, was also noted the other study [2].

Research conducted on the faecal metabolome [51], the absence of clinical signs, and the identified microbial diversity results, support the good health status of the free-roaming giraffe included in the current study. A variety of 21 dominant prokaryotic ASVs (more than 1% of total sequences) were identified, most of which could only be classified to unknown ASV (23%) (Fig. 1b). Ruminococcaceae UCG 014 (11%), Ruminococcaceae UCG 005 (7%), *Acinetobacter* (8%), *Treponema 2* (5%) and *Escherichia* / *Shigella* (5%) were the most dominant identified ASVs (Fig. 1b). The order of dominance differs from previous work, in which *Prevotella* (6%) [15], *Treponema* (12%) [28] and again *Treponema* (24%) [33], had the highest calculated identified microbial abundance. These differences in microbiome structure seem to be driven by differences in diets, geographical location, and environment [8]. Zoo-housed animals are only exposed to a limited variety of browse when compared to their free-roaming counterparts. Wild giraffes are browsers, spending almost half of their time dedicated to finding vegetation rich in crude protein and lignin contents [15, 52, 53] from available NAV. The current study acted as first attempt in addressing previous research limitations such as zoo-housed animals, different populations at different locations, feeding practice differences and seasonal influences over time on the same individual [28, 54]. Building on prior research into the microbial composition and variability of giraffes’ gut microbiota [2, 28], the 26 faecal samples collected and analysed in the current study were deemed independent of sampling efforts. As such, they are considered a reliable basis for describing giraffe gut microbiome diversity. Caution should, however, be taken when comparing microbiome characteristics from different studies, as the implemented methodologies may vary too much for accurate deductions to be made [28].

Ruminococcaceae UCG 014 and *Treponema 2* were found to be significantly (*P* < 0.05) higher in relative abundance for giraffes receiving SF (Table 2). *Escherichia* / *Shigella*, *Romboutsia* and *Ruminococcus 1* were found to be significantly (*P* < 0.05) lower in relative abundance for giraffes receiving SF (Table 2). It is also worth noting that significant differences (*P* < 0.05) in the metabolome of the same faecal samples were calculated such as increased levels of amino acid-related (threonine, 5-oxoproline, serine) and organic-related (squalene, napthalene) compounds for giraffes receiving SF, compared to the NAV subgroup [51]. These differences may be attributed to the increased manufactured SF provided at Locations 1, 4 and 5. Additional dietary supplements (SF) available from the commercially manufactured game pellets contained a mixture of forage-, grain- and roughage products, sugar byproducts, proteins, minerals and trace mineral-vitamin [39, 40]. Lesser digestible plant structural material such as protein, cellulose and hemicellulose are present in NAV (compared to SF) but an increased variety in NAV community structures [55] (McDonald et al. 2010) also contribute to changes in the rumen microbiome composition and activity [56].

Higher relative abundance of ASVs such as *Acinetobacter*, *Arthrobacter*, *Bacillus*, *Escherichia* / *Shigella*, *Methanobrevibacter*, *Romboutsia*, Ruminococcaceae UCG 013 and *Treponema 2*, were noted for the wet season, compared to the dry season, whilst lower relative abundance was calculated for *Alistipes*, Prevotellaceae UCG 004, Rikenellaceae RC9 and Ruminococcaceae UCG 010 (Table 2, Fig. 4). No differences in relative abundance were, however, recorded as being significant (Table 2). These differences can be attributed to the seasonal availability of increased digestible forage material, such as new plant shoots and flowers [5, 57] during the growth (wet) season [55, 58]. An increase in sample size and lesser budget constraints would most definitely aid in improving statistical significance of seasonal microbiome profile changes and are noted as some of the limitations of the current study.

Variations in bacterial profiles were also identified for the rumen microbiome composition from faecal samples analysed from the six males and seven females (Fig. 2c). Non-significant (*P* > 0.05) differences, such as higher amounts of *Enterococcus*, *Escherichia* / *Shigella*, *Romboutsia* and lower relative abundance of *Alistipes*, *Arthrobacter*, *Bacillus* and *Treponema 2* (Table 2), were noted for females when compared to males (Fig. 5). Although the gestation time for the giraffe females were not determined for this study, these different microbiome compositions could be indicative of an increased metabolic rate, known for lactating and pregnant female giraffes [59]. Two unique factors that would drive an increased metabolic rate in female giraffes are that they are usually impregnated whilst still lactating and their reproductive cycle is not bounded by seasons [60].

## Conclusions

This is the first study to investigate the rumen microbiome composition present in faecal droppings from healthy, free-roaming giraffes. Research findings indicate significant differences between the prokaryotic composition of faeces analysed from adult, wild giraffe populations receiving SF compared to populations only browsing NAV. Differences in the relative abundance of ASVs occurred between the wet and the dry seasons and variations in bacterial profiles were identified for the rumen microbiome composition of the two different giraffe sexes. This suggests that faecal prokaryotic analysis can be used an additional non-invasive monitoring technique to effectively predict changes in populations’ nutritional status, animal health issues and ultimately contribute to preventing diet-related deaths in giraffes.

## Supplementary Information

Below is the link to the electronic supplementary material.

Supplementary file1 (DOCX 264 KB)

## Data Availability

The datasets generated during and/or analysed during the current study are available from the corresponding author on reasonable request.

## References

[1] Bekele W, Zegeye A, Simachew A, Assefa G (2021) Functional metagenomics from the rumen environment—a review. ABB 12:125–141. 10.4236/abb.2021.125009

[2] Li A, Liu B, Li F, He Y, Wang L, Fakhar-e-Alam Kulyar M, Li H, Fu Y, Zhu H, Wang Y et al (2021) Integrated bacterial and fungal diversity analysis reveals the gut microbial alterations in diarrheic giraffes. Front Microbiol 12:712092. 10.3389/fmicb.2021.712092 34475863 10.3389/fmicb.2021.712092 PMC8406688

[3] Hobson PN, Stewart CS (eds) (2012) The rumen microbial ecosystem, 2nd edn. Springer, Dordrecht

[4] Henderson G, Cox F, Ganesh S, Jonker A, Young W et al (2015) Rumen microbial community composition varies with diet and host, but a core microbiome is found across a wide geographical range. Sci Rep 5:14567. 10.1038/srep14567 26449758 10.1038/srep14567 PMC4598811

[5] Mitchell G (2021) How giraffes work. Oxford University Press, New York

[6] Youngblut ND, Reischer GH, Walters W, Schuster N, Walzer C, Stalder G, Ley RE, Farnleitner AH (2019) Host diet and evolutionary history explain different aspects of gut microbiome diversity among vertebrate clades. Nat Commun 10:2200. 10.1038/s41467-019-10191-3 31097702 10.1038/s41467-019-10191-3 PMC6522487

[7] Giebelhaus RT, Nguyen G, Schmidt SA, Wang S, Mesfin EY, Nam SL, De La Mata AP, Harynuk JJ (2024) GC×GC-TOFMS analysis of fecal metabolome stabilized using an at-home stool collection device. Appl Biosci 3:348–359. 10.3390/applbiosci3030023

[8] Ley RE, Hamady M, Lozupone C, Turnbaugh PJ, Ramey RR, Bircher JS, Schlegel ML, Tucker TA, Schrenzel MD, Knight R et al (2008) Evolution of mammals and their gut microbes. Science 320:1647–1651. 10.1126/science.1155725 18497261 10.1126/science.1155725 PMC2649005

[9] Codron D, Codron J (2009) Reliability of δ13C and δ15N in faeces for reconstructing savanna herbivore diet. Mamm Biol 74:36–48. 10.1016/j.mambio.2007.12.005

[10] Leslie DM, Bowyer RT, Jenks JA (2008) Facts from feces: nitrogen still measures up as a nutritional index for mammalian herbivores. J Wildl Manag 72:1420–1433. 10.2193/2007-404

[11] Ley RE, Lozupone CA, Hamady M, Knight R, Gordon JI (2008) Worlds within worlds: evolution of the vertebrate gut microbiota. Nat Rev Microbiol 6:776–788. 10.1038/nrmicro1978 18794915 10.1038/nrmicro1978 PMC2664199

[12] Pannoni SB, Proffit KM, Holben WE (2022) Non-invasive monitoring of multiple wildlife health factors by fecal microbiome analysis. Ecol Evol 12:1–12. 10.1002/ece3.8564 10.1002/ece3.8564 PMC8826075 35154651

[13] Simon J-C, Marchesi JR, Mougel C, Selosse M-A (2019) Host-microbiota interactions: from Holobiont theory to analysis. Microbiome 7:5. 10.1186/s40168-019-0619-4 30635058 10.1186/s40168-019-0619-4 PMC6330386

[14] Clauss M, Rose P, Hummel J, Hatt J-M (2006) Serous fat atrophy and other nutrition-related health problems in captive giraffe (*Giraffa camelopardalis*)—an evaluation of 83 necropsy reports

[15] Roggenbuck M, Sauer C, Poulsen M, Bertelsen MF, Sørensen SJ (2014) The giraffe (*Giraffa camelopardalis*) rumen microbiome. FEMS Microbiol Ecol 90:237–246. 10.1111/1574-6941.12402 25087453 10.1111/1574-6941.12402

[16] Schmidt DA, Ball RL, Grobler D, Ellersieck MR, Griffin ME, Citino SB, Bush M (2007) Serum concentrations of amino acids, fatty acids, lipoproteins, vitamins A and E, and minerals in apparently healthy, free-ranging southern giraffe (*Giraffa camelopardalis* Giraffe). Zoo Biol 26:13–25. 10.1002/zoo.20114 19360558 10.1002/zoo.20114

[17] McAloose D, Colegrove KM, Newton AL (2018) Chapter 1—Wildlife necropsy. Pathology of wildlife and zoo animals. Elsevier, p 30

[18] van der Walt MS, Daffue W, Goedhals J, Serfontein C, Deacon F (2023) A preliminary study on the functionality of the carotid-vertebral anastomotic artery in the regulation of blood flow in the giraffe (*Giraffa camelopardalis*) by duplex ultrasound examination. Int J Zool 2023:1–9. 10.1155/2023/8395360

[19] Orellana C, Parraguez VH, Arana W, Escanilla J, Zavaleta C, Castellaro G (2020) Use of fecal indices as a non-invasive tool for nutritional evaluation in extensive-grazing sheep. Animals 10:46. 10.3390/ani10010046 10.3390/ani10010046 PMC7022733 31881641

[20] Erben V, Poschet G, Schrotz-King P, Brenner H (2021) Evaluation of different stool extraction methods for metabolomics measurements in human faecal samples. BMJNPH 4:374–384. 10.1136/bmjnph-2020-000202 10.1136/bmjnph-2020-000202 PMC8718864 35028509

[21] Gregor R, Probst M, Eyal S, Aksenov A, Sasson G, Horovitz I, Dorrestein PC, Meijler MM, Mizrahi I (2022) Mammalian gut metabolomes mirror microbiome composition and host phylogeny. ISME J 16:1262–1274. 10.1038/s41396-021-01152-0 34903850 10.1038/s41396-021-01152-0 PMC9038745

[22] Jain A, Li XH, Chen WN (2019) An untargeted fecal and urine metabolomics analysis of the interplay between the gut microbiome, diet and human metabolism in Indian and Chinese adults. Sci Rep 9:9191. 10.1038/s41598-019-45640-y 31235863 10.1038/s41598-019-45640-y PMC6591403

[23] Cersosimo LM, Lachance H, St-Pierre B, Van Hoven W, Wright A-DG (2015) Examination of the rumen bacteria and methanogenic archaea of wild impalas (Aepyceros Melampus Melampus) from Pongola. South Africa Microb Ecol 69:577–585. 10.1007/s00248-014-0521-3 25351144 10.1007/s00248-014-0521-3

[24] Kartzinel TR, Hsing JC, Musili PM, Brown BRP, Pringle RM (2019) Covariation of diet and gut microbiome in African Megafauna. Proc Natl Acad Sci USA 116:23588–23593. 10.1073/pnas.1905666116 31685619 10.1073/pnas.1905666116 PMC6876249

[25] Bahrndorff S, Alemu T, Alemneh T, Lund Nielsen J (2016) The microbiome of animals: implications for conservation biology. Int J Genomics 2016:1–7. 10.1155/2016/5304028 10.1155/2016/5304028 PMC4852354 27195280

[26] Muller Z (2016) The IUCN red list of threatened species: *Giraffa camelopardalis*, Giraffe, Errata Version

[27] Leuthold BM, Leuthold W (1972) Food habits of giraffe in Tsavo National Park, Kenya. East African Wildlife J 10:129–141. 10.1111/j.1365-2028.1972.tb01173.x

[28] AlZahal O, Valdes EV, McBride BW (2016) Analysis of the distal gut bacterial community by 454-pyrosequencing in captive giraffes (*Giraffa camelopardalis*): distal gut bacterial community in captive giraffe. Zoo Biol 35:42–50. 10.1002/zoo.21252 26584008 10.1002/zoo.21252

[29] Dagg AI (2014) Giraffe: biology behaviour and conservation. Cambridge University Press

[30] David LA, Maurice CF, Carmody RN, Gootenberg DB, Button JE, Wolfe BE, Ling AV, Devlin AS, Varma Y, Fischbach MA et al (2014) Diet rapidly and reproducibly alters the human gut microbiome. Nature 505:559–563. 10.1038/nature12820 24336217 10.1038/nature12820 PMC3957428

[31] Hummel J, Clauss M, Zimmermann W, Johanson K, Nørgaard C, Pfeffer E (2005) Fluid and particle retention in captive Okapi (*Okapia Johnstoni*). Comp Biochem Physiol A: Mol Integr Physiol 140:436–444. 10.1016/j.cbpb.2005.02.006 15936703 10.1016/j.cbpb.2005.02.006

[32] Mitchell G, Roberts DG, van Sittert SJ (2015) The digestive morphophysiology of wild, free-living, giraffes. Comp Biochem Physiol A: Mol Integr Physiol 187:119–129. 10.1016/j.cbpa.2015.05.015 26021980 10.1016/j.cbpa.2015.05.015

[33] Schmidt JM, Henken S, Dowd SE, McLaughlin RW (2018) Analysis of the microbial diversity in the fecal material of giraffes. Curr Microbiol 75:323–327. 10.1007/s00284-017-1383-y 29085995 10.1007/s00284-017-1383-y

[34] Xi L, Song Y, Qin X, Han J, Chang Y-F (2021) Microbiome analysis reveals the dynamic alternations in gut microbiota of diarrheal *Giraffa camelopardalis*. Front Vet Sci 8:649372. 10.3389/fvets.2021.649372 34124218 10.3389/fvets.2021.649372 PMC8192810

[35] Groussin M, Mazel F, Sanders JG, Smillie CS, Lavergne S, Thuiller W, Alm EJ (2017) Unraveling the processes shaping mammalian gut microbiomes over evolutionary time. Nat Commun 8:14319. 10.1038/ncomms14319 28230052 10.1038/ncomms14319 PMC5331214

[36] Kamble A, Sawant S, Singh H (2020) 16S ribosomal RNA gene-based metagenomics: a review. Biomed Res J 7:5. 10.4103/BMRJ.BMRJ_4_20

[37] Mucina L, Rutherford MC (2006) The vegetation of South Africa, Lesotho and Swaziland, Vol. Strelitzia 19. South African National Biodiversity Institute: Pretoria

[38] Leslie DM, Starkey EE (1985) Fecal indices to dietary quality of cervids in old-growth forests. J Wildl Manag 49:142. 10.2307/3801860

[39] Voermol Game Pellet Composition (2024) Voermol. https://voermol.co.za/wpcontent/uploads/2018/07/Game_Pellets_100.pdf

[40] Wild G Lubern Normal Game Pellet Composition (2024) Lubern Voere. https://www.lubern.co.za/wpcontent/uploads/2019/02/Lubern-Booklet2-2_Page_45.jpg

[41] Wrench JM, Meissner HH, Grant CC, Casey NH (1996) Environmental factors that affect the concentration of P and N in faecal samples collected for the determination of nutritional status. Koedoe 39:1–6

[42] Instruction manual: ZymoBIOMICS DNA MiniPrep Kit version 1.5.4 (2023)

[43] Bolyen E, Rideout JR, Dillon MR, Bokulich NA, Abnet CC, Al-Ghalith GA, Alexander H, Alm EJ (2019) Reproducible, interactive, scalable and extensible microbiome data science using QIIME 2. Nat Biotechnol 37:852–857. 10.1038/s41587-019-0209-9 31341288 10.1038/s41587-019-0209-9 PMC7015180

[44] Martin M (2011) Cutadapt removes adapter sequences from high-throughput sequencing reads. EMBnet.journal 17:10–12. 10.14806/ej.17.1.200

[45] Callahan BJ, McMurdie PJ, Rosen MJ, Han AW, Johnson AJA, Holmes SP (2016) DADA2: high-resolution sample inference from Illumina Amplicon data. Nat Methods Brief Commun 13:1–7. 10.1038/nMeth.3869 10.1038/nmeth.3869 PMC4927377 27214047

[46] Quast C, Pruesse E, Yilmaz P, Gerken J, Schweer T, Yarza P (2013) The SILVA ribosomal RNA gene database project: improved data processing and web-based tools. Nucleic Acids Res 41:D590–D596. 10.1093/nar/gks1219 23193283 10.1093/nar/gks1219 PMC3531112

[47] Dhariwal A, Chong J, Habib S, King IL, Agellon LB, Xia J (2017) MicrobiomeAnalyst: a web-based tool for comprehensive statistical, visual and meta-analysis of microbiome data. Nucleic Acids Res 45:W180–W188. 10.1093/nar/gkx295 28449106 10.1093/nar/gkx295 PMC5570177

[48] Pang Z, Lu Y, Zhou G, Hui F, Xu L, Viau C, Spigelman AF, MacDonald PE, Wishart DS, Li S et al (2024) MetaboAnalyst 60: towards a unified platform for metabolomics data processing, analysis and interpretation. Nucleic Acids Res 52:W398–W406. 10.1093/nar/gkae253 38587201 10.1093/nar/gkae253 PMC11223798

[49] Excel Microsoft 365 Office (2016) Excel Data Analysis ToolPak (Version 2405) [Computer software]. Microsoft.

[50] Du Preez I, Loots DT (2013) New sputum metabolite markers implicating adaptations of the host to mycobacterium tuberculosis, and vice versa. Tuberculosis 93:330–337. 10.1016/j.tube.2013.02.008 23477940 10.1016/j.tube.2013.02.008

[51] Grobbelaar A, Osthoff G, Du Preez I, Deacon F (2024) First insights into the fecal metabolome of healthy, free-roaming giraffes (*Giraffa camelopardalis*): an untargeted GCxGC/TOF-MS metabolomics study. Metabolites 14:586. 10.3390/metabo14110586 39590822 10.3390/metabo14110586 PMC11596133

[52] Caister LE, Shields WM, Gosser A (2003) Female tannin avoidance: a possible explanation for habitat and dietary segregation of giraffes (*Giraffa camelopardalis peralta*) in Niger. Afr J Ecol 41:201–210. 10.1046/j.1365-2028.2003.00422.x

[53] Shipley LA, Blomquist S, Danell K (1998) Diet choices made by free-ranging moose in northern Sweden in relation to plant distribution, chemistry, and morphology. Can J Zool 76:1722–1733. 10.1139/z98-110

[54] Caporaso JG, Lauber CL, Costello EK, Berg-Lyons D, Gonzalez A, Stombaugh J, Knights D, Gajer P, Ravel J, Fierer N et al (2011) Moving pictures of the human microbiome. Genome Biol 12:R50. 10.1186/gb-2011-12-5-r50 21624126 10.1186/gb-2011-12-5-r50 PMC3271711

[55] McDonald P, Edwards RA, Greenhalgh JFD, Morgan CA, Sinclair LA, Wilkinson RG (2010) Animal nutrition, 7th edn. Harlow, Pearson

[56] Kinyamario JI, Macharia JNM (1992) aboveground standing crop, protein content and dry matter digestibility of a tropical grassland range in the Nairobi National Park, Kenya. African J Ecol 30:33–41. 10.1111/j.1365-2028.1992.tb00476.x

[57] Dagg AI, Foster JB (1976) The giraffe: its biology, behaviour and ecology. Van Nostrand Reinhold Co., New York

[58] Janecke BB, Smit GN (2015) Faecal nitrogen of browser and mixed feeder game species during different seasons. African J Range Forage Sci 32:203–212. 10.2989/10220119.2014.959055

[59] del Castillo SM, Bashaw MJ, Patton ML, Rieches RR, Bercovitch FB (2005) Fecal steroid analysis of female giraffe (*Giraffa camelopardalis*) reproductive condition and the impact of endocrine status on daily time budgets. Gen Comp Endocrinol 141:271–281. 10.1016/j.ygcen.2005.01.011 15804514 10.1016/j.ygcen.2005.01.011

[60] Bercovitch FB, Bashaw MJ, Del Castillo SM (2006) Sociosexual behavior, male mating tactics, and the reproductive cycle of giraffe *Giraffa camelopardalis*. Horm Behav 50:314–321. 10.1016/j.yhbeh.2006.04.004 16765955 10.1016/j.yhbeh.2006.04.004

