# Supplementary material for: The Faecal Microbiome Analysed from Healthy, Free-Roaming Giraffes (Giraffa camelopardalis)
Source: Curr Microbiol. 2025 Feb 24;82(4):151. doi: 10.1007/s00284-025-04127-y (PMC11850562; doi:10.1007/s00284-025-04127-y)
Supplement: Supplementary file 1 — Supplementary file1 (DOCX 264 KB) [file 284_2025_4127_MOESM1_ESM.docx]

**Appendix**

**The faecal microbiome analysed from healthy, free-roaming giraffes (*Giraffa camelopardalis*)**

Andri Grobbelaar ^1^, Gernot Osthoff ^2^, Francois Deacon ^1^ and Errol D. Cason ^1^*

^1^ Department of Animal Sciences, Faculty of Natural and Agricultural Sciences. University of the Free State. PO Box 339, Bloemfontein, 9300, SA; andri.giraffe@gmail.com; casoned@ufs.ac.za; deaconf@ufs.ac.za

^2^ Department of Microbiology and Biochemistry, Faculty of Natural and Agricultural Sciences. University of the Free State. PO Box 339, Bloemfontein, 9300, SA; osthoffg@ufs.ac.za

* Correspondence: CasonED@ufs.ac.za; Tel.: (+27)514012606

The following supporting information is available (other data sets can be made available by the authors on reasonable request.):


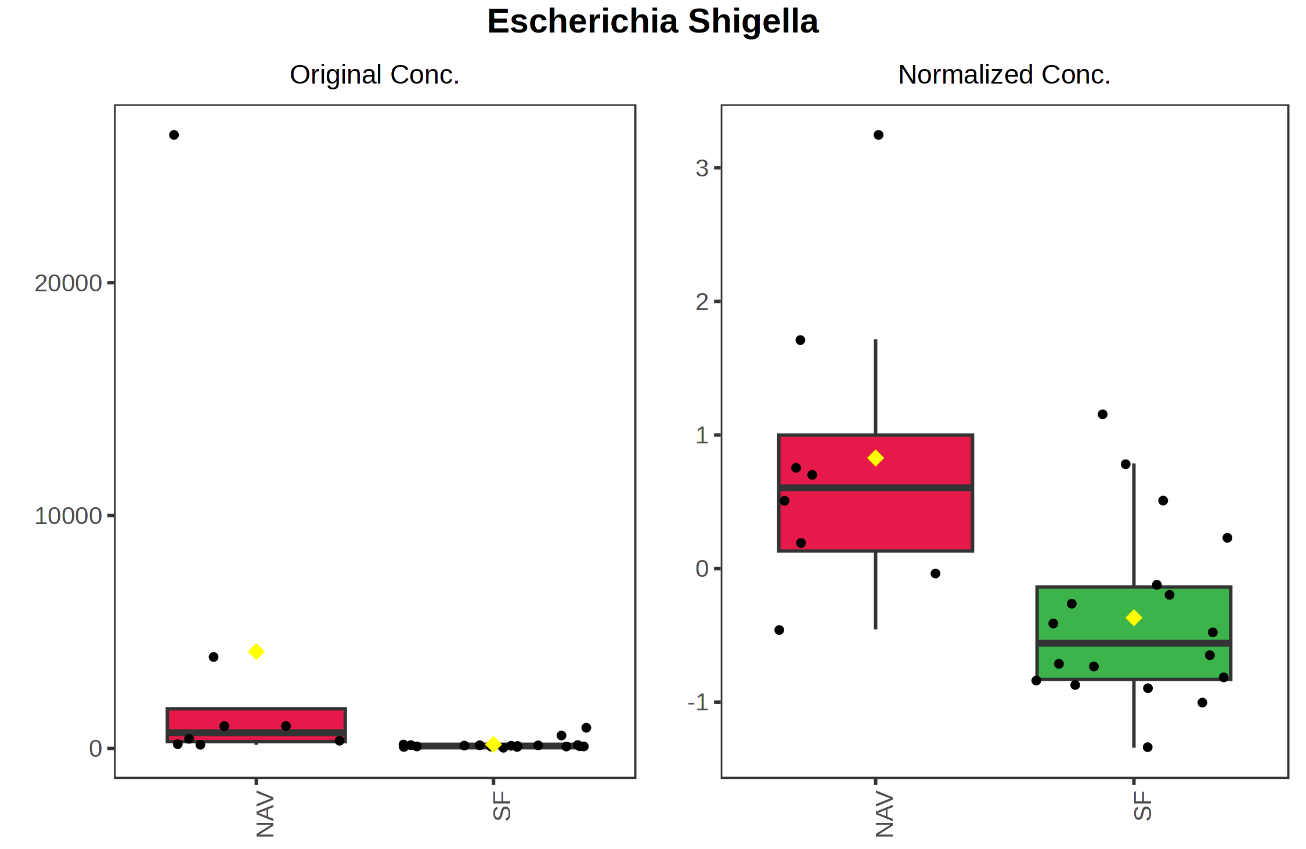


**Fig. 1** Box-whisper plot illustrating the significant changes (p < 0.05) in *Escherichia* / *Shigella* relative abundance identified from the faecal droppings from giraffe receiving supplemental feed (SF) or only natural available vegetation (NAV) from locations in the Free State Province, South Africa


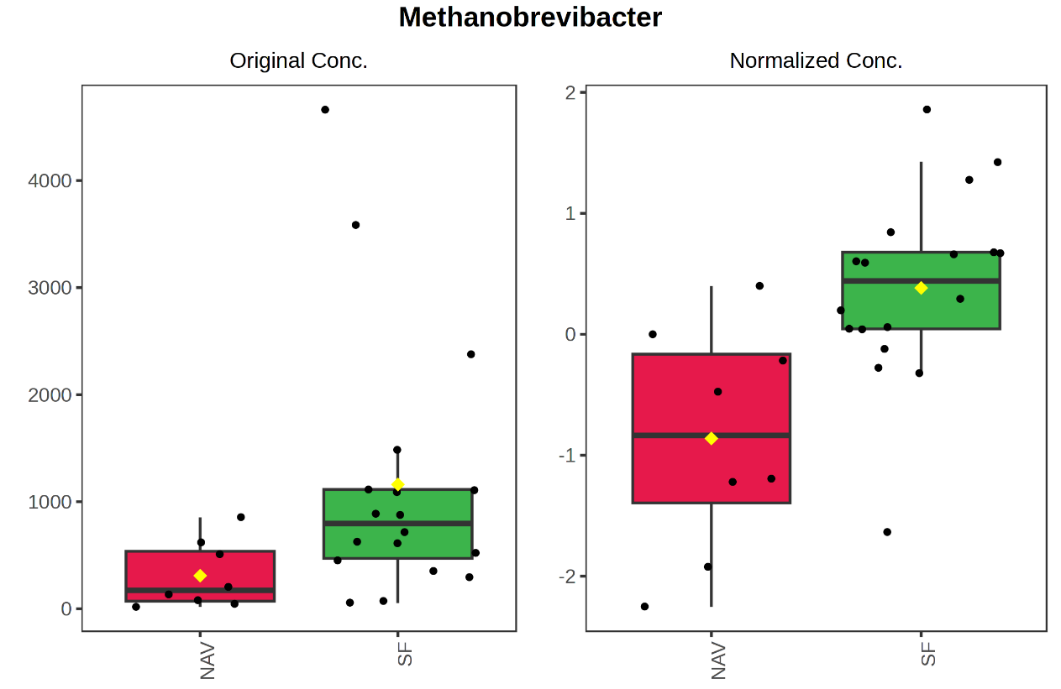


**Fig. 2** Box-whisper plot illustrating the significant changes (p < 0.05) in *Methanobrevibacter* relative abundance identified from the faecal droppings from giraffe receiving supplemental feed (SF) or only natural available vegetation (NAV) from locations in the Free State Province, South Africa


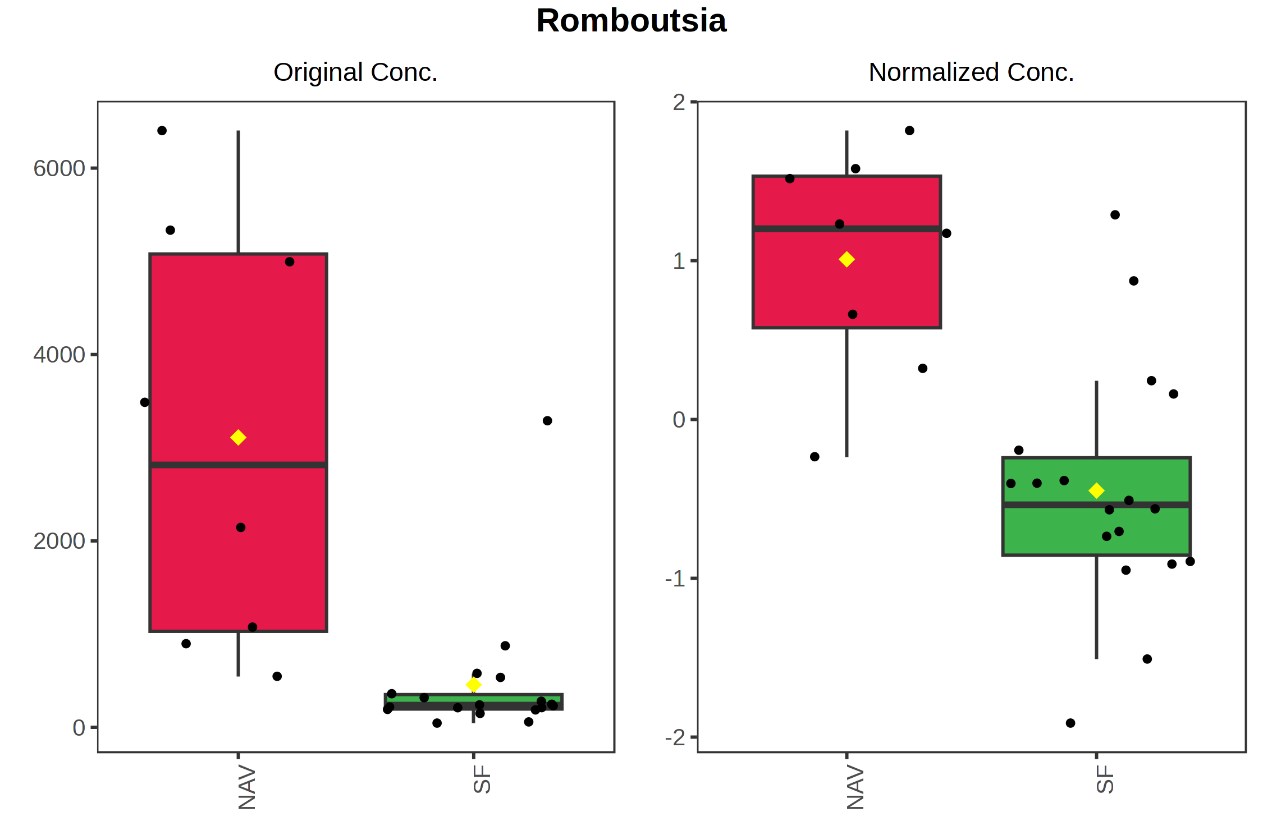


**Fig. 3** Box-whisper plot illustrating the significant changes (p < 0.05) in *Romboutsia* relative abundance identified from the faecal droppings from giraffe receiving supplemental feed (SF) or only natural available vegetation (NAV) from locations in the Free State Province, South Africa


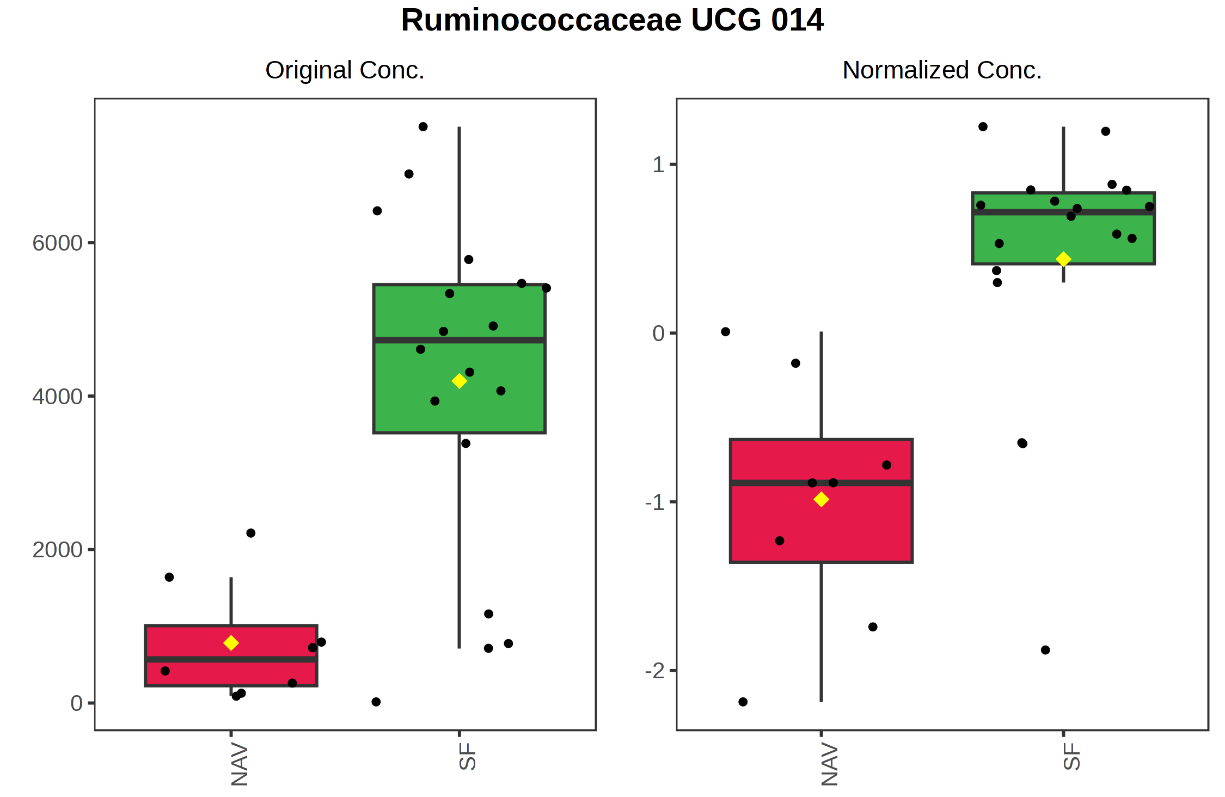


**Fig. 4** Box-whisper plot illustrating the significant changes (p < 0.05) in Ruminococcaceae UCG 014 relative abundance identified from the faecal droppings from giraffe receiving supplemental feed (SF) or only natural available vegetation (NAV) from locations in the Free State Province, South Africa


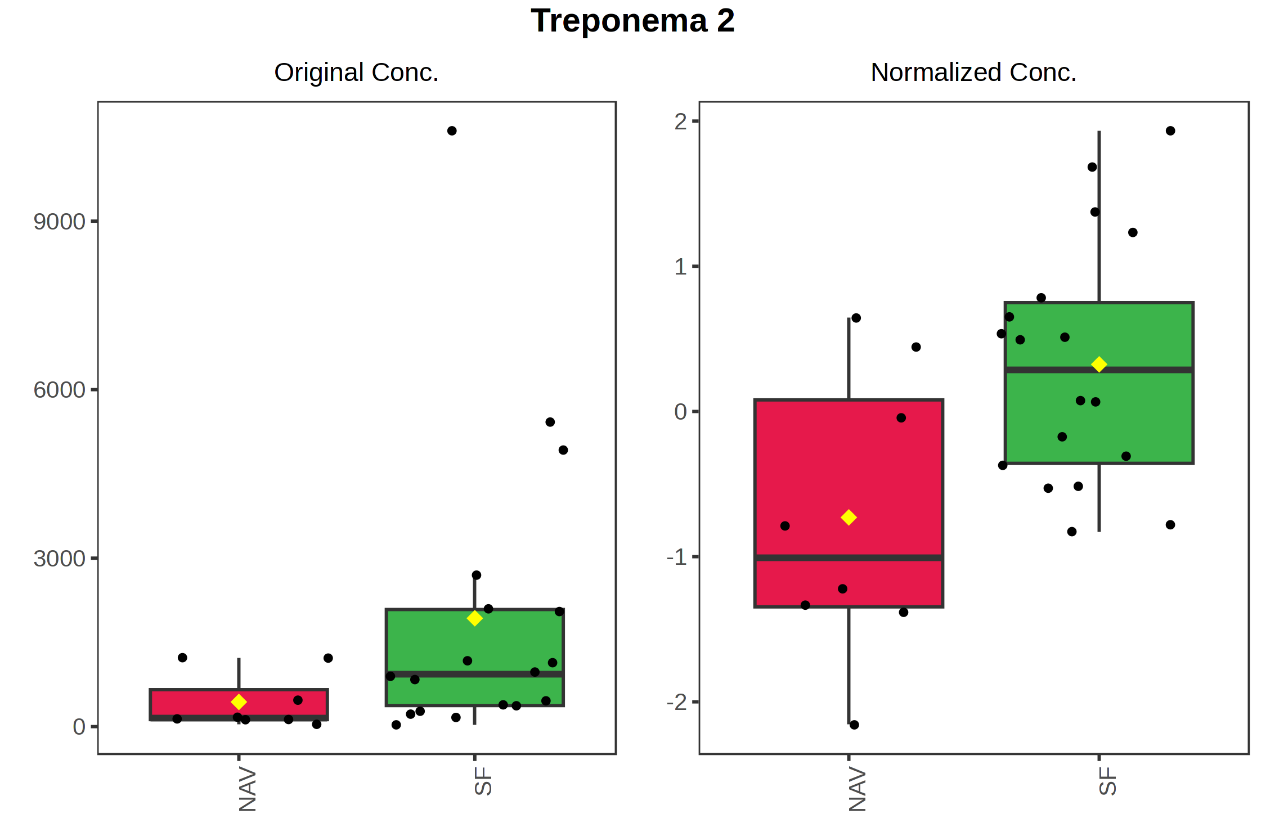


**Fig. 5** Box-whisper plot illustrating the significant changes (p < 0.05) in *Treponema* 2 relative abundance identified from the faecal droppings from giraffe receiving supplemental feed (SF) or only natural available vegetation (NAV) from locations in the Free State Province, South Africa
